# 80N as the Optimal Assistive Threshold for Wearable Exoskeleton-Mediated Gait Rehabilitation in Parkinson’s Disease: A Prospective Biomarker Validation Study

**DOI:** 10.3390/healthcare13070799

**Published:** 2025-04-02

**Authors:** Xiang Wei, Jian Sun, Guanghan Lu, Jingxuan Liu, Jiuqi Yan, Xiong Wei, Hongyang Cai, Bei Luo, Wenwen Dong, Liang Zhao, Chang Qiu, Wenbin Zhang, Yang Pan

**Affiliations:** 1 Department of Functional Neurosurgery, The Affiliated Brain Hospital of Nanjing Medical University, 264 Guangzhou Road, Nanjing 210029, China; yaoxiaocc@stu.njmu.edu.cn (X.W.); sunjian266@stu.njmu.edu.cn (J.S.); luguanghan@stu.njmu.edu.cn (G.L.); laixs@stu.njmu.edu.cn (J.L.); yanjiuqi@stu.njmu.edu.cn (J.Y.); beiluo@stu.njmu.edu.cn (B.L.); dongww1020@njmu.edu.cn (W.D.); zhaoliang@njmu.edu.cn (L.Z.); cyrusqiu@stu.njmu.edu.cn (C.Q.); 2 Department of Geriatrics, The Affiliated Brain Hospital of Nanjing Medical University, Nanjing 210029, China; weixiong@stu.njmu.edu.cn (X.W.); chy0512@stu.njmu.edu.cn (H.C.)

**Keywords:** wearable device, lower limb exoskeletons, Parkinson’s disease, gait disturbance, gait monitoring

## Abstract

**Background and Objectives**: Robotic exoskeletons show potential in PD gait rehabilitation. But the optimal assistive force and its equivalence to clinical gold standard assessments are unclear. This study aims to explore the clinical equivalence of the lower limb exoskeleton in evaluating PD patients’ gait disorders and find the best assistive force for clinical use. **Methods**: In this prospective controlled trial, 60 PD patients (Hoehn and Yahr stages 2–4) and 60 age-matched controls underwent quantitative gait analysis using a portable exoskeleton (Relink-ANK-1BM) at four assistive force levels (0 N, 40 N, 80 N, 120 N). Data from 57 patients and 57 controls were analyzed with GraphPad Prism 10. Different statistical tests were used based on data distribution. **Results**: ROC analysis showed that exoskeleton-measured velocity had the strongest power to distinguish PD patients from controls (AUC = 0.9198, *p* < 0.001). Other parameters also had high reliability and validity. There was a strong positive correlation between UPDRS-III lower extremity sub-score changes and gait velocity changes in PD patients (r = 0.8564, *p* < 0.001). The 80 N assistive force led to the best gait rehabilitation, with a 58% increase in gait velocity compared to unassisted walking (*p* < 0.001). **Conclusions**: 80 N is the optimal assistive threshold for PD gait rehabilitation. The wearable lower limb exoskeleton can be an objective alternative biomarker to UPDRS-III, enabling personalized home-based rehabilitation.

## 1. Introduction

Gait impairment is a characteristic manifestation in mid-to-late-stage Parkinson’s disease (PD). Epidemiological studies show that over 80% of PD patients experience gait and postural instability during disease progression [1]. The characteristic motor phenotypes include three main aspects: (1) reduced velocity related to bradykinesia, (2) increased stride-to-stride variability and asymmetry, and (3) impaired postural control mechanisms [2]. These features together significantly deteriorate quality of life and contribute to disability in advanced PD patients [3,4]. Emerging evidence implicates nigrostriatal dopaminergic neurodegeneration as the principal pathophysiological substrate. The depletion of dopaminergic transmission reduces cortical excitability, thereby disrupting basal ganglia–brainstem–spinal cord regulatory pathways [5]. This neural circuit dysfunction ultimately compromises automatic movement modulation, mechanistically underpinning the emergence of gait and postural abnormalities. Current clinical assessments for gait and postural disturbances rely on timed tests (e.g., 10MWT, TUG) and scales (e.g., Berg Balance Scale), which are objective but task specific and lack continuous spatiotemporal data [6,7]. This lack of temporal and spatial specificity, along with objectivity, can lead to misjudgments that affect disease evaluation and treatment intervention [8,9]. Therefore, there is an urgent need for an objective means to assess the levels of gait and postural disturbances in order to guide rehabilitation and improve long-term outcomes for patients.

Nearly all patients diagnosed with Parkinson’s disease undergo long-term exercise rehabilitation [10], which includes a variety of aerobic and anaerobic activities, such as treadmill training, balance exercises, cycling, swimming, and yoga [11,12]. Studies have indicated that exercise rehabilitation not only improves gait and postural balance disturbances in patients with Parkinson’s disease [13,14,15] but also positively affects non-motor symptoms, including depression, anxiety, and cognitive impairment [16]. The literature suggests that the underlying physiological mechanisms may be related to neuroprotection [17] and synaptic plasticity [18]. However, with the gradual development of artificial intelligence, traditional physical rehabilitation methods are increasingly being supplemented and, in some cases, replaced by AI-driven approaches [19,20,21]. Compared to traditional physical rehabilitation models, artificial intelligence offers advantages such as resource efficiency, convenience, accuracy, objectivity, and the ability to monitor patients’ gait over extended periods [22,23,24]. A randomized controlled trial by Gryfe et al. [25] demonstrated that robot-assisted gait interventions improved gait endurance (6-min walk test) compared to traditional therapy, highlighting the potential of robotic devices in PD rehabilitation. This highlights the advantages and effectiveness of robotic lower limb exoskeletons in PD rehabilitation. However, the high cost of these rehabilitation robots limits their use to clinical settings, making home monitoring and rehabilitation challenging for some PD patients, and they are generally unsuitable for most patients receiving outpatient medication adjustments and regular follow-ups.

Lower limb exoskeletons (LLexo), built on the foundations of artificial intelligence and machine learning [26,27], are equipped with sensors and actuators at various joints to continuously record gait data during patient training. By providing varying levels of assistance, these devices help patients improve their gait and assist with walking, offering a more accessible and portable rehabilitation solution [28,29]. This wearable LLexo represents a paradigm shift in neurorehabilitation. It offers portability (2.1 kg per leg) and affordability (less than 5% of the cost of clinical robots) while maintaining therapeutic efficacy, thereby enabling future home-based rehabilitation training.

In summary, this study utilized a more lightweight, portable, and cost-effective home-based lower limb exoskeleton robot rehabilitation training system. This system not only assisted in rehabilitation training but also detected the patients’ gait parameters and provided guidance for their rehabilitation and prognosis. We adopted a mode that combined the lower limb exoskeleton robot with assistive forces, aiming to: (1) further validate the reliability and validity of the clinical application of the wearable lower limb exoskeleton robot; (2) establish research on the wearable exoskeleton as an alternative biomarker for UPDRS-III; and (3) systematically quantify the assistive force threshold for PD gait rehabilitation.

## 2. Materials and Methods

### 2.1. Clinical Material

This study included 60 Parkinson’s disease (PD) patients with gait disturbances who visited the Department of Functional Neurosurgery, The Affiliated Brain Hospital of Nanjing Medical University, from September 2023 to September 2024, alongside 60 age-matched healthy controls. The PD patients constituted the experimental group, while the healthy controls formed the control group. The inclusion criteria for the experimental group were: (1) a diagnosis of idiopathic PD with a disease duration of less than 10 years; (2) Hoehn and Yahr stages of 2.0–4.0; (3) a levodopa challenge test improvement rate greater than 30%; (4) Mini-Mental State Examination (MMSE) score > 23 and Montreal Cognitive Assessment (MoCA) score > 22; (5) the presence of gait and postural disturbances with the exclusion of other causes; and (6) the ability to cooperate with the exoskeleton assistive system and follow-up. The exclusion criteria included: (1) severe mental or psychological disorders or cognitive impairments that hindered cooperation and follow-up; (2) severe gait and postural disturbances preventing normal ambulation; and (3) changes in PD medication, physical dysfunction, lower limb sensory deficits, vestibular disorders, or other neurological or orthopedic conditions involving the lower limbs, as well as cardiovascular comorbidities during the study. This research was conducted in accordance with the Declaration of Helsinki and received ethical approval from the Ethics Committee of Nanjing Medical University Affiliated Brain Hospital (Approval No.: 2022-KY005-01). All patients provided written informed consent. This study was registered in the Chinese Clinical Trial Registry (ChiCTR2200065799). Three patients from each group were excluded due to non-cooperation.

General information is shown in Table 1 below.

### 2.2. Method

#### 2.2.1. Pre-Gait Training Preparations

Before testing, participants were allocated to either the experimental or control group. During the study, patients in the experimental group were required to maintain their regular antiparkinsonian medication regimen. On the same day, they underwent UPDRS-III motor assessments and gait training with the lower limb exoskeleton in both off- and on-medication states. No participant had received any form of rehabilitation treatment or participated in any clinical trials outside the study protocol in the three months prior to the study. All patients in the experimental group were responsive to levodopa, with an improvement rate greater than 30% during the levodopa challenge test. Patients were randomized (1:1) via computer-generated sequence. The off state was confirmed ≥12 h post-levodopa, and the on state after 1.5× maintenance dose. Blinded clinicians performed the UPDRS-III evaluations.

#### 2.2.2. Exoskeleton System Operation and Assistance Mechanisms

**Figure 1:**
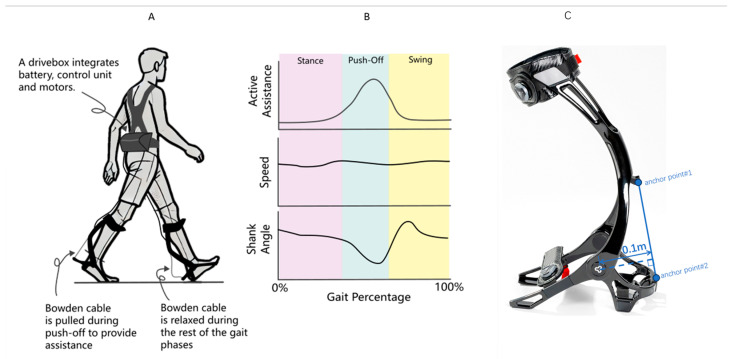
(**A**) Schematic diagram of the lower limb exoskeleton robot, featuring adjustable assistive devices on both feet. (**B**) Gait parameters detected during patient walking, illustrating the variations in gait parameters throughout the gait cycle. (**C**) The torque applied to the ankle joint by the exoskeleton in this study is generated by Bowden cables and an ankle joint spring. The Bowden cables apply force at two anchor points on the leg brace, with the ankle joint as the pivot point. The moment arm length is constant at 0.1 m, and its variation during the entire plantarflexion rotation is negligible. Additionally, the tensile force from the Bowden cables must overcome the elastic force of the ankle joint spring. The torque applied to the ankle joint can be expressed as: $\tau_{a}=f\cdot R-\tau_{s}$, where f = tensile force of the Bowden cable (measured in real time by tension sensors at the leg brace anchor points); R = moment arm length (constant at 0.1 m); and $\tau_{s}$ = torque of the ankle joint spring, approximated by $\tau_{s}=\tau_{0}+k\cdot \theta_{a}$*,* where $\tau_{0}$ = initial torque (approximately −2.033 N·m), k = torsional stiffness (approximately 0.033 N·m/°), and $\theta_{a}$= ankle plantarflexion angle.

The exoskeleton system (Relink-ANK-1BM, Yuan Ye Technology, Suzhou, China) employs soft robotic technology with enhanced carbon fiber ankle-foot orthoses (AFOs) replacing conventional fabric-based calf wraps, providing superior mechanical support during weight-bearing ambulation. This wearable apparatus integrates four core components (Figure 1): (1) a textile-based interface with embedded Bowden cables spanning the bilateral lower extremities; (2) dual carbon fiber AFOs that generate ankle plantarflexion torque through cable retraction (0–180 N programmable assistance); (3) inertial measurement units (IMUs) sampling at 100 Hz for real-time kinematic tracking (shank displacement, ankle angle, spatiotemporal gait parameters); and (4) load cells that quantify human–robot interaction forces at the cable terminals. The moment arm between the force application point of the exoskeleton and the ankle joint is 10 cm (0.1 m). The torque for different assistive forces can be calculated using the formula: Torque = Assistive Force × Moment Arm [30]. The exoskeleton system was utilized by placing the lower limbs into the exoskeleton, and securing the device with magnetic locks and straps. An ergonomic harness positioned the drive unit at the patient’s waist, connected via flexible traction lines to the foot exoskeleton (Figure 1A). The accompanying software facilitated force feedback control and gait data recording (see product manual) (Figure 1B).

#### 2.2.3. Monitoring Paradigms and Gait Parameter Collection with the Exoskeleton

First, the participants performed three 10 m walks at a self-selected speed on a pressure-sensitive mat (GAITRite^®^) synchronized with the exoskeleton data to validate the spatiotemporal accuracy. Subsequently, after the patient put on the lower limb exoskeleton robot, he/she carried out an adaptive activity for 10 min. Patients stood at the starting point and adapted to the exoskeleton before monitoring commenced as they initiated walking. Participants were instructed to walk at a steady and as-fast-as-possible pace to the 10 m endpoint. The primary function of the exoskeleton robot was to monitor gait in PD patients, capturing parameters such as velocity, cadence, stride, stance phase ratio, and calf ankle angle. The experimental group received progressively increasing assistance (0, 40, 80, and 120 N), with the gait parameters recorded for each level of assistance, while the control group was monitored at 0 N (no assistance). All testing occurred in a dedicated exercise rehabilitation room, with 5 to 10 min of rest between phases based on patient need. Each assistance level was repeated three times, and the average value was recorded.

### 2.3. Observation Indicators and Evaluation Methods

A comprehensive assessment was conducted on the gait parameters recorded by the lower limb exoskeleton system under different assistance levels (0, 40, 80, and 120 N) in the experimental group, alongside the 0 N parameters from the control group. The evaluation of gait disturbances included velocity (m/s), cadence (steps/min), stride(m), stance phase ratio (%), and calf ankle angle (°), with stride, stance phase ratio, and calf ankle angle recorded separately for the left and right sides. Velocity represents the distance covered in a unit of time, while cadence indicates the number of footfalls per minute. Stride is the longitudinal distance between two consecutive heel strikes on the same side, equivalent to the sum of left and right strides. The stance phase ratio (%) is calculated as the time the lower limb is in contact with the ground and bearing weight divided by the total gait cycle duration, multiplied by 100%. The calf ankle angle measures the range of motion at the knee and ankle joints during flexion and extension.

**Figure 2:**
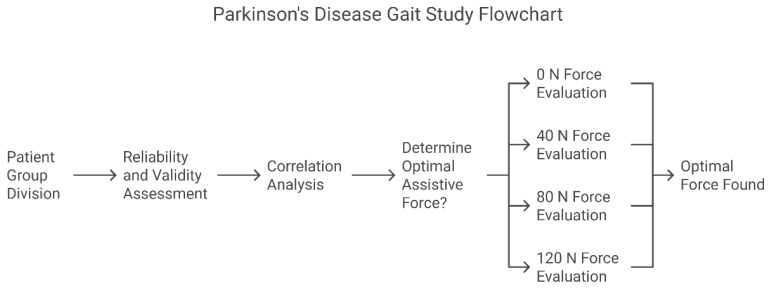
Sixty patients were enrolled in the PD experimental group and sixty in the healthy control group (three patients were excluded from each group due to not meeting the inclusion criteria).

Stratified validation research design: First, the measurement reliability and validity were established through ROC analysis (Step 1). Subsequently, the clinical correlation analysis was verified (Step 2). Finally, the optimal assistive force parameters were determined through a randomized intervention (Step 3). (Figure 2) The statistical significance threshold was set at α = 0.05 (Bonferroni correction).

Step 1. Reliability and Validity Assessment: Compare the spatiotemporal gait parameters of the PD experimental group and the healthy control group with an assistive force of 0 N to determine their reliability and validity;

Step 2. Correlation Analysis: Compare the gait parameters of the PD experimental group recorded by the lower limb exoskeleton with UPDRS-III lower limb scores to determine their clinical correlation;

Step 3. Determination of the Optimal Assistive Force Threshold: Compare the gait parameters of the PD experimental group under different assistive forces (0/40/80/120 N) to determine the optimal assistive force.

### 2.4. Statistical Methods

Statistical analyses were performed using GraphPad Prism 10 (GraphPad, San Diego, CA, USA). A normality test was conducted on the samples; data meeting normal distribution were expressed as the mean ± standard deviation (x ± s), while data that did not meet normal distribution were presented as the median and interquartile range. For intergroup comparisons of normally distributed data, either a two-sample *t*-test or one-way ANOVA was applied. Non-parametric tests were used for comparisons of non-normally distributed data. A *p*-value of less than 0.05 was considered indicative of statistical significance.

## 3. Results

### 3.1. Reliability and Validity Test

**Figure 3:**
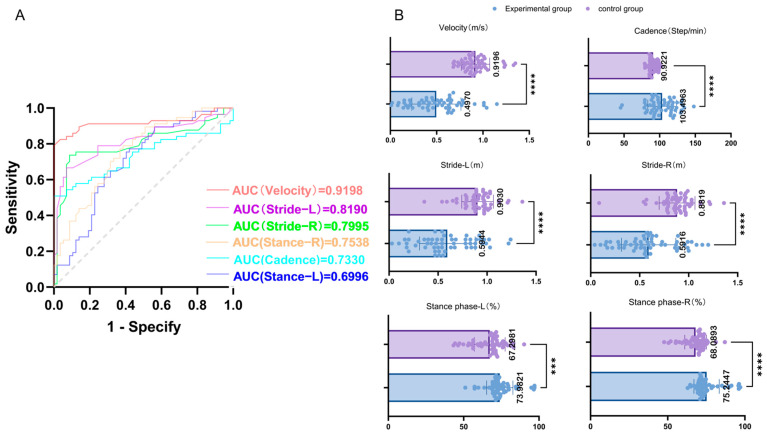
(**A**) Area under the curve (AUC) for different gait parameters: The AUCs for various gait parameters (velocity, cadence, left and right strides, and left and right stance phases) are presented. A larger AUC indicates higher reliability and validity. As shown in the figure, velocity has the largest AUC, indicating it possesses the highest reliability and validity among the assessed parameters. (**B**) Scatter plots of gait parameter comparison between experimental and control groups: The average values of the gait parameters for the experimental and control groups are summarized above. The symbols indicate the statistical significance of differences between the groups: *** represents *p* < 0.001, and **** represents *p* < 0.0001.

The spatiotemporal gait parameters of the PD experimental group (***n*** = 57) and the healthy control group (***n*** = 57) under 0 N assistive force were compared using the receiver operating characteristic (ROC) curve. The area under the curve (AUC) for velocity was 0.9198 (*p* < 0.001), for cadence it was 0.7370 (*p* < 0.001), for left stride length it was 0.8580, and for right stride length it was 0.8734 (both *p* < 0.001). The AUC for the left stance phase was 0.6915, and for the right stance phase it was 0.7371 (both *p* < 0.05). The results are shown in Table 2. The above results indicated that the lower limb exoskeleton had significant reliability and validity in monitoring patients with PD gait disorders. Specifically, the walking speed, cadence, stride length, and proportion of the stance phase all exhibited high reliability and validity, among which walking speed was the most powerful discriminative factor between the PD group and the control group (AUC = 0.9198, *p* < 0.001). The relevant ROC curves and scatter plots are shown in Figure 3.

### 3.2. Clinical Correlation Analysis

**Figure 4:**
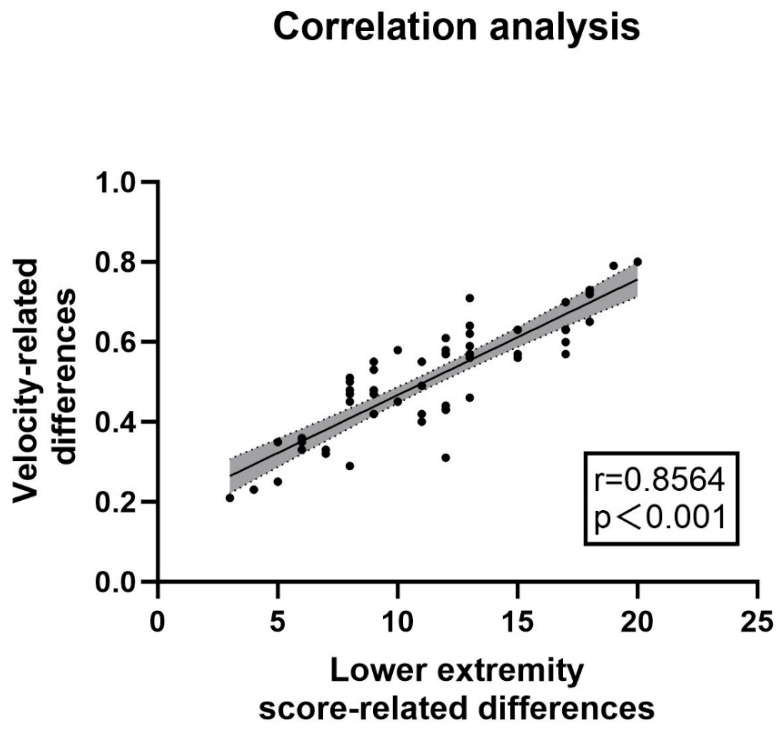
Correlation between velocity changes and UPDRS-III lower limb score changes: The analysis of the correlation between changes in velocity and differences in UPDRS III lower limb gait sub-scores was conducted using data from the lower limb exoskeleton robot. The correlation coefficient was found to be r = 0.8564 (*p* < 0.001), indicating a strong relationship. The accompanying figure illustrates this correlation, while the relevant sub-scores from UPDRS III include items 3d, 3e, 5d, 5e, 9a, and 9b, as detailed in the appendix. These findings suggested that monitoring with the lower limb exoskeleton can effectively substitute for UPDRS-III lower limb gait sub-scores, demonstrating its utility in assessing gait characteristics in patients with Parkinson’s disease.

The clinical correlation was verified by comparing the gait parameters and UPDRS-III scores between the “off” state and the “on” state in the PD experimental group (n = 57). (1) Scale Results: Among the patients included in the experimental group, there were 33 males and 24 females. The average age of the patients was 62 years old, and the average disease duration was 6 years. The average UPDRS-III score in the “off” state was 46.18, while in the “on” state it was 22.23, with an average improvement rate of more than 51.77%. (2) Spatiotemporal Gait Parameters Measured by the Lower Limb Exoskeleton: The average velocity of the patients increased from 0.41 m/s to 0.94 m/s. The left stride length increased from 0.59 m to 0.68 m, and the right stride length increased from 0.59 m to 0.69 m. The proportion of the left stance phase decreased from 72.34% to 70.46%, and that of the right stance phase decreased from 73.70% to 68.25%. Increases in walking speed and stride length, as well as a decrease in the proportion of the stance phase, all indicated an improvement in the patients’ gait ability, and all of these changes were statistically significant (*p* < 0.05). The relevant parameters are shown in Table 3. A correlation analysis was conducted between the difference in UPDRS-III scale scores before and after the impact test and the difference in velocity. The results showed a strong correlation (r = 0.8564, *p* < 0.001, see Figure 4). The above results suggested that the lower limb exoskeleton robot can be effectively used to monitor the gait changes of patients and has the potential to serve as a substitute for UPDRS-III as a potential biomarker.

### 3.3. Determination of Optimal Assistive Force Parameters Through Random Intervention

**Figure 5:**
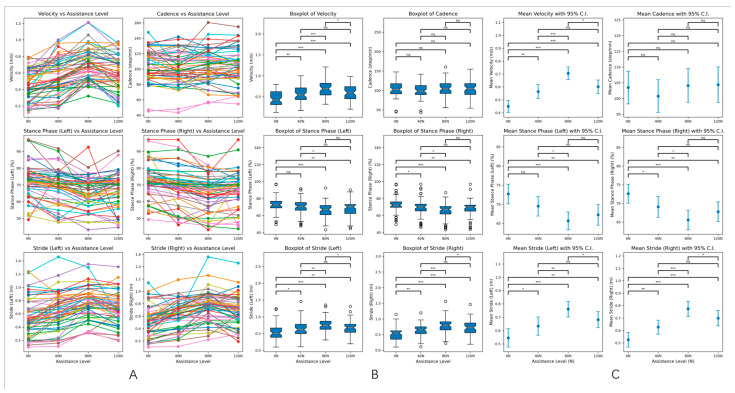
(**A**). Line graphs of velocity, cadence, stride, and stance phase ratio under different assistance levels. The line graphs display the changes in velocity, cadence, stride, and stance phase ratio for the 57 patients in the experimental group at various assistance levels. This visualization illustrates the trends and patterns in gait parameters as external assistance is varied. (**B**). Box plots of gait parameters under different assistance levels. The box plots provide a summary of the gait parameters for the experimental group at different assistance levels. Each plot displays the median, quartiles, and potential outliers, offering a clear view of the distribution and variability of the data for velocity, stride, and stance phase percentages. (**C**). The 95% confidence intervals for gait parameters under different assistance levels. The figure shows the 95% confidence intervals for the gait parameters of the experimental group across different assistance levels. These intervals indicate the range within which we can be 95% confident that the true mean of each parameter lies, highlighting the reliability of the observed differences. In the context of statistical significance: none of * indicates *p* > 0.05 (ns: not significant), * indicates *p* < 0.05 (significant), ** indicates *p* < 0.01 (highly significant), *** indicates *p* < 0.001 (very highly significant).

Different assistive force modes (0, 40, 80, 120 N) were applied to the PD experimental group, and a random intervention was carried out to determine the optimal assistive force mode for the gait rehabilitation of PD patients. The results showed that there were significant statistical differences in velocity, stride length, and the proportion of the stance phase in the PD experimental group under different assistive forces (0, 40, 80, 120 N), with *p* < 0.05. However, there was no significant difference in cadence under different assistive forces, with *p* > 0.05 (as shown in Table 4 below). In addition, according to the results of statistical analysis, the average values of the patients’ gait parameters were optimized best under the 80 N assistive force, suggesting that 80 N is the optimal assistive threshold for PD gait rehabilitation (Figure 5). Additionally, considering that differences in patients’ body mass index (BMI) and gender may have certain effects on powered exoskeleton gait rehabilitation, we stratified the experimental group according to BMI and gender based on the optimal improvement in walking speed at 80 N to explore the impact of BMI and gender on assistance, thereby eliminating potential biases (as shown in Tables S1 and S2).

## 4. Discussion

Previous studies suggested that gait disturbances in PD patients primarily result from stiffness and limited mobility in the hip and knee joints, leading to abnormal gait function, with a notably increased proportion of the stance phase. Additionally, factors such as trunk flexion, lack of dorsiflexion at the ankle, and abnormal limb swing contribute to reductions in velocity, cadence, and stride [31]. After establishing the feasibility of using the lower limb exoskeleton robot, this study objectively measured the gait parameters of the experimental group and the healthy control group. ROC analysis revealed significant differences in velocity, cadence, stride, and stance phase ratio between PD and healthy individuals (*p* < 0.05). Specifically, PD patients exhibited reduced velocity, cadence, and stride, alongside an increased proportion of the stance phase, consistent with previous research. Moreover, the largest AUC (AUC = 0.9198, *p* < 0.001) was observed for velocity, suggesting that velocity can serve as the optimal indicator for evaluating the severity of gait disorders in PD patients. That is, objectively measuring the walking speed through the lower limb exoskeleton robot can objectively assess the gait conditions of patients and determine the severity of their condition.

The lower limb exoskeleton system (LLexo) has emerged as a more accessible and cost-effective rehabilitation device, and it is gradually being applied in assisted rehabilitation of various clinical conditions, including but not limited to spinal cord injuries, acquired brain injuries [32,33], and movement disorders such as PD [34]. However, its feasibility and reliability still require further investigation [35]. The results of this study demonstrate a significant correlation between the change in velocity measured by the lower limb exoskeleton robot and UPDRS-III lower limb scores, with a correlation coefficient of r = 0.8564. This suggests that the assessment by the lower limb exoskeleton system has significant clinical equivalence and holds the potential to serve as a substitute for UPDRS-III as a potential biomarker, consistent with previous research findings [36,37]. The introduction of wearable lower limb exoskeleton robots may fundamentally transform assessment and rehabilitation strategies for Parkinson’s disease. Unlike traditional timed walk tests (e.g., 10 MWT, TUG), exoskeleton systems provide dynamic feedback and multi-parameter analysis (e.g., joint kinematics, stance phase symmetry), thereby supporting personalized assessment and rehabilitation. Their portability also enables home-based monitoring, effectively addressing the limitations of clinic-dependent assessments. Moreover, traditional physical rehabilitation, guided by therapists, is not only resource-intensive but also faces challenges in sustaining long-term recovery due to the nature of the disease. By contrast, wearable lower limb exoskeletons effectively address these issues with their lightweight design, affordability, and strong objectivity, enabling home rehabilitation and monitoring for patients with gait disturbances [38,39]. Additionally, visual assessment methods that assist in determining the severity of movement disorders in Parkinson’s disease are becoming increasingly widespread. Patients perform specific movements and walk a designated distance in open environments, with videos subsequently transmitted for automatic analysis of their motor deficits using machine learning. This approach allows for efficient and objective evaluation of the patient’s condition [40,41]. In the future, the combination of lower limb exoskeletons and visual assessments may enhance the accuracy and objectivity of evaluating motor symptoms in Parkinson’s disease patients. This integration is likely to significantly advance the development of remote diagnosis and treatment for PD and other movement disorders [42].

The mechanisms underlying gait disturbances in PD patients remain highly controversial. A randomized controlled trial by Nathalie Chastan MD et al. [43] indicated that among 15 Parkinson’s disease (PD) patients with freezing of gait, velocity and stride were significantly lower compared to 17 patients without this condition. This suggests that freezing of gait may be related to overall gait disturbances in PD, and improving this symptom could enhance gait performance. In our study, we employed a powered lower limb exoskeleton robot to address freezing of gait by providing external assistance. Unlike medications such as levodopa that directly affect central nervous pathways to enhance initiation, this approach allows for direct manipulation of the force applied, minimizing drug-related side effects while exploring optimal initiation forces. Our analysis revealed significant statistical differences in velocity, stride, and stance phase percentages under 40, 80, and 120 N assistance compared to no assistance (0 N) (*p* < 0.05), indicating improved gait capabilities with external stance. However, cadence did not show significant differences across assistance levels (*p* > 0.05), consistent with previous findings that gait rhythm in PD patients typically remains unchanged or increases as a compensatory mechanism for reduced stride. This suggests a minimal relationship between gait disturbances and decreased cadence [44,45]. Additionally, intergroup comparisons of gait parameters under different assistance levels revealed that velocity and stride were maximized, while the stance phase percentage was minimized at 80 N assistance. This suggests that the gait parameters are optimal at 80 N, indicating that this level may represent the best assistance for rehabilitation in Parkinson’s disease patients. The possible reason is that the 80 N threshold may correspond to the minimum assistive torque (~15–20 Nm, 0.5 m/s) required to overcome PD-related stiffness of the hip flexors [46].Furthermore, considering that the assistive force magnitude of this exoskeleton may be influenced by factors such as patient BMI (e.g., heavier patients may require greater assistance) and gender (e.g., males, with potentially greater muscle mass than females might need higher force levels), we conducted subgroup analyses for BMI-related effects on gait velocity improvements across different force levels and gender-stratified comparisons. The BMI analysis indicated that at lower assistance levels (40 N), BMI showed a significant correlation with assistive force requirements (*p* < 0.05), likely because minimal assistance merely provided external support without effectively improving gait rhythm, thus making body weight a dominant factor. However, as the assistive force increased, the influence of BMI diminished. Notably, no significant correlation was observed between BMI and the optimal assistive threshold (80 N) (*p* > 0.05). This suggests that 80 N assistance sufficiently restores natural gait cycles, effectively addressing pathological gait patterns in Parkinson’s disease (PD) patients. Meanwhile, the gender subgroup analysis pointed out that both male and female subgroups exhibited maximal gait velocity improvements at 80 N, indicating gender-independent efficacy of this force level. In summary, we propose that the assistive threshold of this exoskeleton is governed by gait kinematics (e.g., joint torque requirements) rather than absolute body weight calibration or gender-specific differences (e.g., muscle strength). Furthermore, its adaptive design dynamically aligns with individual joint angles and gait patterns. At the optimal assistive force level (80 N), it effectively restores physiological gait cycles, thereby ameliorating gait disturbances in Parkinson’s disease patients.

The preliminary results of this study indicate that using a lower limb exoskeleton robot system as an objective assessment tool for gait disturbances is a feasible and effective method. The powered exoskeleton has shown significant benefits in improving gait function, particularly at an assistance level of 80 N. However, this study has several limitations: (1) Sample size and generalizability: Currently, the sample size of this study is relatively small, lacking the support of large-sample data. There may be some clinical biases. In the future, a large number of samples and multi-center clinical studies are still needed for further verification. (2) The moment arm in this study was limited to 10 cm, so the conclusions may not be applicable to other types of exoskeletons. Future research is needed to develop various moment arms compatible with human anatomy and collect gait parameters to enhance comparability. Additionally, the assistive force pattern of the exoskeleton was fixed; integrating PID control to dynamically adjust assistance based on spatiotemporal gait parameters should be prioritized. (3) Absence of individualized training protocols: A motion capture system should be implemented in future studies to synchronously measure joint kinematics and force application points, thereby obtaining dynamic moment arms throughout the gait cycle and accurately measuring dynamic gait parameters. (4) This study used limited gait parameters (velocity, cadence, step length, stance phase ratio, etc.) to draw conclusions, lacking sufficient persuasiveness. Future work should synchronously collect additional kinematic, kinetic, and electromyography (EMG) data to observe dynamic changes during the entire gait cycle for comprehensive rehabilitation evaluation and support.

To comprehensively evaluate the feasibility and effectiveness of the lower limb exoskeleton system in PD patients, as well as the varying impacts of different assistance levels on gait, larger-scale clinical trials are needed. These should involve a larger sample size and more extensive data collection. Additionally, implementing individualized rehabilitation treatment plans and long-term training monitoring is crucial to determine the long-term prognostic effects of the exoskeleton on gait recovery in PD patients, which may require further advancements in sensor systems and machine learning algorithms.

Moreover, beyond rehabilitation efficacy, cost effectiveness must be considered to assess the viability and economic impact of this portable lower limb exoskeleton in both clinical and home settings.

## 5. Conclusions

In summary, gait disturbances in Parkinson’s disease (PD) patients correlate with poor long-term prognoses, making long-term rehabilitation stance crucial. While the application of artificial intelligence in rehabilitation has shown promising results, its future development faces significant challenges. There is a need for more precise data processing algorithms, potentially through the integration of various machine learning techniques, expansion of databases, or the use of deep learning methods, to enhance the accuracy and clinical applicability of lower limb exoskeleton robots.

## Figures and Tables

**Table 1:** Demographic data of the experimental and control groups.

Group	Experimental Group	Control Group	*p*
Age (years)	61.82 ± 10.01	61.63 ± 8.91	>0.05
Duration of disease (years)	6 (5, 7)	/	/
M-H&Y	3.28 (2.76, 3.90)	/	/
UPDRS-III (OFF)	46.18 ± 12.63	/	/
UPDRS-III (ON)	22.23 ± 6.71	/	/
LCT Improvement Rate (%)	51.77 ± 7.39	/	/

M-H&Y, Modified Hoehn and Yahr Scale; UPDRS-III, Unified Parkinson’s Disease Rating Scale Part III; LCT, levodopa challenge test. Data meeting normal distribution are expressed as mean ± standard deviation (x ± s), while data that did not meet normal distribution are presented as median and interquartile range.

**Table 2:** Gait parameters: experimental group vs. control group.

	Limb Side	Experimental Group	Control Group	*p*
Velocity (m/s)	/	0.50 ± 0.27	0.89 (0.82, 0.99)	****
Cadence (steps/min)	/	103.50 ± 20.10	90.92 ± 5.96	****
Stride (m)	Left	0.59 ± 0.28	0.91 (0.82, 0.98)	****
	Right	0.59 ± 0.28	0.89 (0.79, 0.97)	****
Stance phase (%)	Left	72.64 (70.41, 76.80)	69.36 (60.61, 72.28)	***
	Right	72.25 (70.63, 76.28)	69.78 (65.28, 71.85)	****

This table presents gait parameter data for the experimental and control groups under no assistance (0 N). The symbols indicate the statistical significance of differences between the groups: *** represents *p* < 0.001, and **** represents *p* < 0.0001.

**Table 3:** Demographic and movement assessment data for the experimental group during “on” and “off” Phases.

			OFF	ON	*p*
Number			57	/	/
Gender (Men/Women)			33/24	/	/
Age (years)			61.82 ± 10.01	/	/
Duration of disease (years)			6.24 (5.17, 7.36)	/	/
UPDRS-III score			46.18 ± 12.63	22.23 ± 6.71	***
LLexo Motion Monitoring	Velocity (m/s)		0.41 (0.31, 0.51)	0.94 (0.87, 0.98)	***
	Stride(m)	Left	0.59 ± 0.28	0.68 ± 0.28	**
		Right	0.59 ± 0.27	0.69 ± 0.29	**
	Stance phase (%)	Left	72.35 (64.65, 78.83)	70.58 (61.03, 75.12)	**
		Right	71.93 (66.57, 75.59)	70.32 (61.11, 73.97)	**

(1) UPDRS III, Unified-Parkinson Disease Rating Scale Part III; LLexo, lower limb exoskeleton; OFF, levodopa challenge test (LCT)-Pre; ON, levodopa challenge test (LCT)-Post; (2) In the context of statistical significance: ** indicates *p* < 0.01 (highly significant), *** indicates *p* < 0.001 (very highly significant). Note: Due to poor data quality, we excluded data with abnormal variations in the proportion of left and right stance phase before and after LCT between the experimental group and the control group in order to ensure the quality and validity of the data.

**Table 4:** Gait parameters of experimental group under different assistance levels.

Parameter		0 N	40 N	80 N	120 N	*p*
Velocity(m/s)	/	0.45 ± 0.18	0.56 ± 0.21	0.71 ± 0.19	0.60 ± 0.20	***
Cadence (Steps/min)	/	103.50 ± 20.10	100.77 ± 20.13	104.11 ± 20.70	104.39 ± 21.77	ns
Stride(m)	Left	0.59 ± 0.28	0.63 ± 0.29	0.68 ± 0.28	0.67 ± 0.29	*
	Right	0.53 ± 0.23	0.63 ± 0.22	0.77 ± 0.23	0.70 ± 0.24	**
Stance phase (%)	Left	72.64 (69.24, 75.89)	70.21 (64.97, 72.47)	64.23 (60.58, 71.29)	68.35 (62.37, 72.34)	**
	Right	72.22 (69.45, 76.51)	70.05 (64.72, 73.55)	67.14 (60.74, 71.08)	69.97 (64.72, 72.31)	***

This table presents the gait parameter data under different assistance levels. For parameters that satisfy normal distribution, values are expressed as mean ± standard deviation. For those that do not meet normal distribution, values are expressed as median and interquartile range. In the context of statistical significance: ns indicates *p* > 0.05 (not significant), * indicates *p* < 0.05 (significant), ** indicates *p* < 0.01 (highly significant), *** indicates *p* < 0.001 (very highly significant).

## Data Availability

The relevant data used in this study are available from the corresponding authors upon request.

## Supplementary Materials

The following supporting information can be downloaded at: https://www.mdpi.com/article/10.3390/healthcare13070799/s1; Table S1. Correlation between Body Mass Index (BMI) and Differences in Velocity under Different Assistive Force Groups; Table S2. Effects of Gender Subgroups on Velocity under Different Assistive Forces.

## Abbreviations

PD	Parkinson’s Disease
UPDRS-III	Unified Parkinson’s Disease Rating Scale Part III
ROC	Receiver Operating Characteristic
AUC	Area Under the Curve
LLexo	Lower Limb Exoskeleton
OFF	Levodopa Challenge Test-Pre
ON	Levodopa Challenge Test-Post
LCT	Levodopa Challenge Test
M-H&Y	Modified Hoehn and Yahr Scale
MMSE	Mini-Mental State Examination
MoCA	Montreal Cognitive Assessment
IMU	Inertial Measurement Unit
AFO	Ankle-Foot Orthosis
BMI	Body Mass Index
PID	Proportional-Integral-Derivative
EMG	Electromyography
10 MWT	10-Meter Walk Test
TUG	Timed Up and Go Test
