# Supplementary material for: 80N as the Optimal Assistive Threshold for Wearable Exoskeleton-Mediated Gait Rehabilitation in Parkinson’s Disease: A Prospective Biomarker Validation Study
Source: Healthcare (Basel). 2025 Apr 2;13(7):799. doi: 10.3390/healthcare13070799 (PMC11989103; doi:10.3390/healthcare13070799)
Supplement: Supplementary file 1 [file healthcare-13-00799-s001.zip › healthcare-3496877-supplementary.pdf]

Table S1. Correlation between Body Mass Index (BMI) and Differences in Velocity under Different Assistive Force Groups

	Weight（Kg）	BMI(Kg/m²)	Δ40N	Δ80N	Δ120N
Experimental Group（n=57）	61.93±7.42	23.26±2.02	0.07(0.02,0.18)	0.23(0.15,0.36)	0.11(0.06,0.25)
Person	/	/	0.343102	0.254592	0.061623
P	/	/	**	NS	NS

Table S1 presents the correlations between patients' BMI and the differences in walking speed under different assistive forces (Δ40N, Δ80N, Δ120N compared to 0N) in the experimental group. The results show an average body weight of 61.93±7.42 kg, mean BMI of 23.26±2.02, Δ40N of 0.07 (0.02, 0.18), Δ80N of 0.23 (0.15, 0.36), Δ120N of 0.11 (0.06, 0.25), with corresponding Pearson correlation coefficients (PCCs) of 0.343102, 0.254592, and 0.061623 respectively. Hypothesis testing revealed significant correlation between △40N and BMI (P＜0.05), while no significant correlations were observed for △80N and △120N (P＞0.05). These findings indicate that at lower assistive force (40N), BMI may influence required exoskeleton assistance - higher BMI patients may need greater assistance. However, at 80N and 120N assistive forces, no significant relationships were found, suggesting BMI does not affect required assistance levels. This demonstrates that the impact of BMI on exoskeleton assistance varies with force magnitude: significant at 40N, unclear at 80N, and negligible at 120N.For data that satisfy normal distribution, values are expressed as mean ± standard deviation. For those that do not meet normal distribution, values are expressed as median and interquartile range. In the context of statistical significance: ns indicates P > 0.05 (not significant)，* indicates P < 0.05 (significant)，** indicates P < 0.01 (highly significant)，*** indicates P < 0.001 (very highly significant)，**** indicates P < 0.0001 (extremely significant).

Table S2. Effects of Gender Subgroups on Velocity under Different Assistive Forces

Gender		0N	40N	80N	120N	P
Men（33）	/	0.44±0.21	0.56±0.23	0.73±0.19	0.58±0.21	****
Women（24）	/	0.46±0.15	0.57±0.20	0.67±0.19	0.63±0.19	****

Table S2 divided the experimental group (n=57) into male (n=33) and female (n=24) subgroups by gender. For data meeting normal distribution, values are presented as mean ± standard deviation. For non-normally distributed data, values are expressed as median and interquartile range. Statistical significance is denoted as follows: ns = P > 0.05 (non-significant), * = P < 0.05 (significant), ** = P < 0.01 (highly significant), *** = P < 0.001 (very highly significant), **** = P < 0.0001 (extremely significant). The table suggests significant differences between male and female subgroups across all assistive force levels (0, 40, 80, 120N). Notably, both genders showed the most pronounced improvement at 80N, indicating that 80N is the optimal assistive force level for exoskeleton rehabilitation in Parkinson's disease, regardless of gender.
